# TNF*α* Promotes Th17 Cell Differentiation through IL-6 and IL-1*β* Produced by Monocytes in Rheumatoid Arthritis

**DOI:** 10.1155/2014/385352

**Published:** 2014-11-11

**Authors:** Yingxia Zheng, Lei Sun, Ting Jiang, Dongqing Zhang, Dongyi He, Hong Nie

**Affiliations:** ^1^Shanghai Institute of Immunology, Institutes of Medical Sciences, Shanghai Jiao Tong University School of Medicine, 280 South Chongqing Road, Shanghai 200025, China; ^2^Department of Clinical Laboratory, Xinhua Hospital, Shanghai Jiao Tong University School of Medicine, 1665 Kongjiang Road, Shanghai 200092, China; ^3^School of Pharmacy, Shanghai Jiao Tong University, 800 Dongchuan Road, Shanghai 200240, China; ^4^Guanghua Integrative Medicine Hospital, 540 Xinhua Road, Shanghai 200052, China; ^5^Institute of Arthritis Research, Shanghai Academy of Chinese Medical Sciences, 540 Xinhua Road, Shanghai 200052, China

## Abstract

TNF*α* plays an important role in autoimmune pathogenesis and is the main therapeutic target of rheumatoid arthritis. However, its underlying mechanism is not completely understood. In this study, we described that Th17 cells were accumulated in synovial fluid, which was attributable to TNF*α* aberrantly produced in rheumatoid synovium. Interestingly, TNF*α* cannot induce IL-17 production of CD4^+^ T cells directly, but through the monocytes high levels of IL-1*β* and IL-6 in a TNFRI and TNFRII dependent manner from the active RA patients are produced. TNF*α* was shown to enhance the phosphorylation level of STAT3 and the expression level of transcription factor RORC of CD4^+^ T cells when cultured with CD14^+^ monocytes. Treatment with an approved TNF*α* blocking antibody showed marked reduction in the levels of IL-6, IL-1*β*, and IL-17 and the expression level of STAT3 phosphorylation in relation to Th17 cell differentiation in patients with rheumatoid arthritis. The study provides new evidence supporting the critical role of TNF*α* in the pathogenic Th17 cell differentiation in rheumatoid arthritis.

## 1. Introduction

Rheumatoid arthritis (RA) is a chronic, progressive, inflammatory systemic disease affecting about 1% of the population [1]. The pathogenesis of this destructive disease was classically viewed as involving two hierarchical systems, inflammation, and autoimmunity, respectively. It targets primarily the synovial tissues in which pathogenic T cells, such as Th1 and Th17 cells, play an important role [2, 3]. Although the etiology of the disease remains unknown, the increasing evidence suggests that tumor necrosis factor *α* (TNF*α*) plays an important role in the induction of tissue destruction and inflammatory processes in RA [4] and as the main therapeutic target [5–7]. TNF*α* possesses a broad spectrum of proinflammatory properties through its activation of the NF-*κ*B pathway and is a multifaceted cytokine critically involved in rheumatoid synovitis. For example, TNF*α* induces the synthesis of proinflammatory cytokines (such as IL-1 and IL-6) and chemokines (such as IL-8, MCP-1, MIP-1*α*, and RANTES) and activates macrophages, which in turn perpetuate the proinflammatory cytokine milieu in autoimmune pathology [8, 9]. In addition, the activation of NF-*κ*B through TNF*α* receptors (TNFR1 and TNFR2) upregulates antiapoptotic proteins, leading to prolonged survival of inflammatory cells and persistent inflammation [10–12]. Infliximab, the anti-TNF*α* monoclonal antibody, neutralizes membrane-bound TNF*α* and soluble TNF*α*, suppresses TNF*α* production by macrophages and lymphocytes, and greatly suppresses inflammation for the RA patients, with approximately two-thirds of patients exhibiting a clinical response to treatment [13, 14].

Cytokines produced by the pathogenic T cells appeared to be involved in the initiation and perpetuation of RA [15]. IL-17 is capable of promoting inflammation by inducing a variety of proinflammatory mediators, including cytokines, chemokines, and other mediators of bone and cartilage destruction in synovial fibroblasts, monocytes, macrophages, and chondrocytes [16]. IL-17 may also contribute directly to joint damage, because it was shown to act synergistically with TNF*α* and/or IL-1*β* to induce cartilage destruction in vitro and in experimental arthritis in vivo [17, 18]. Th17 cells are thought to arise from naïve T cells primed with IL-6 and TGF-*β* and require continued IL-23 signaling for survival and maintenance [19–21]. It has been reported that activated monocytes from both healthy controls and RA patients induce Th17 responses in an IL-1*β*/TNF*α*-dependent fashion in vitro and activated monocytes from the site of inflammation in RA induce increased Th17 responses in a cell-contact dependent manner in vivo [22]. In particular, dendritic cells and macrophages are shown to promote Th17 cell differentiation through IL-6 and JAK/STAT-3 signaling [23, 24]. There are preliminary indications that Th17 cells are resistance to suppression by Treg cells and the Th17 level is elevated in rheumatoid synovium and correlates with inflammatory parameters in RA patients [25–28]. In the mouse collagen induced arthritis model, IL-17 effect was dependent on the presence of TNF*α* at the early phase, whereas at a later stage the disease was mostly IL-17 driven, which is TNF*α* independent [29].

Our study showed that the production of IL-17 by stimulated CD4^+^ T cells, which is associated with active inflammation, was significantly elevated in RA patients, especially from the synovial fluid mononuclear cells (SFMC). In addition, the production of IL-17 by synovial fluid (SF) from RA patients exposed to anti-TNF*α* in vitro was greatly reduced, and the Th17 transcription factor STAT3 and RORC in T cells was also reduced. Moreover, TNF*α* promoted Th17 cell differentiation through IL-6 and IL-1*β* produced by monocytes in active RA patients. Patients with active RA that response to anti-TNF*α* therapy produced less Th17 cells than the pretreatment. These data suggest that TNF*α* promotes Th17 cell differentiation through monocytes that produce high levels of IL-6 and IL-1*β* in active RA and inhibition of IL-17 by anti-TNF*α* therapy may protect RA patients from severe inflammation.

## 2. Materials and Methods

### 2.1. Patients and Specimens

A total of 40 RA patients were included in the study. All patients fulfilled the American College of Rheumatology criteria (ACR) for RA. The average age of this cohort of patients was 56.7 ± 8.5. They included 35 females and 5 males with disease duration of 11.5 ± 9.5 years. Among the patients, 87.5% were rheumatoid factor positive. The mean ± standard deviation (SD) of erythrocyte sedimentation rate (ESR) was 55.5 ± 34.8 mm/h, and the mean ± SD of C-reactive protein (CRP) was 43.3 ± 42.2 mg/dL. Patients were not under immunosuppressive agents and received nonsteroidal anti-inflammatory drugs during the 2 months before sample collection. Blood specimens were obtained from a group of 36 healthy individuals matched for sex ratio and mean age with the patient group. Synovial fluid from RA patients was centrifuged at 400 g for 5 minutes, and supernatants were collected and immediately stored at –80°C until use. Mononuclear cells were prepared from synovial fluid and blood specimens (SFMC and PBMC) by Ficoll-Hypaque centrifugation (Amersham Biosciences) and were immediately processed for cell culture. A group of ten clinically definite RA patients was treated with infliximab (3 mg/kg infliximab in 250 mL 0.9% NaCl was administered i.v. at weeks 0, 2, 6, 14, and 22) and paired blood specimens were obtained at baseline and 22 weeks after treatment for further analysis. Informed consent was obtained from all study subjects prior to sample collection. The study protocol was approved by the Medical Ethics Review Board of Guanghua Integrative Medicine Hospital.

### 2.2. Cell Purification

CD4^+^ T cells and CD14^+^ monocytes were purified from PBMCs by CD4^+^ T Cell Isolation Kit II and CD14 microbeads (Miltenyi Biotec), according to the manufacturer's instructions, respectively. The purity of cells was greater than 95%.

### 2.3. Th17 Cell Differentiation and Antibody Blocking Experiments

As for the Th17 cell differentiation, FACS-sorted CD4^+^CD45RA^+^CD25^−^ naïve T cells were stimulated with anti-CD3/CD28 antibodies (1 *μ*g/mL, eBioscience), cultured with IL-6 (30 ng/mL, R&D Systems), IL-1*β* (10 ng/mL, R&D Systems), anti-IFN*γ* (10 *μ*g/mL, eBioscience), and anti-IL-4 (10 *μ*g/mL, eBioscience) for 5 days. For antibody blocking experiments, RA-derived synovial fluid was preincubated for 2 h with antibodies directed at IL-6, TNF*α*, IL-1*β*, TGF*β*, TNFRI, and TNFRII, respectively, or an isotype control antibody (R&D Systems) prior to use in cell culture. In the case of IL-23 and IL-21, antibodies against receptor fusion proteins (R&D Systems) were used under the same experimental conditions.

### 2.4. Flow Cytometry

PBMCs from healthy individuals were prepared and stained with CD14 and TNFRI or TNFRII (R&D Systems). For intracellular cytokine staining, cells were incubated for 5 h with PMA (50 ng/mL, Sigma-Aldrich) and ionomycin (0.5 *μ*g/mL, Sigma-Aldrich) or with lipopolysaccharides (LPS, 100 ng/mL, Sigma-Aldrich) in the presence of GolgiPlug (BD Biosciences). The resulting cells were first surface-stained with anti-human CD4 antibody (BD Biosciences) or CD14 antibody (eBioscience) and then stained with antibodies to human IFN*γ* (BD Biosciences), IL-17, IL-6, or IL-1*β* (eBioscience) by using Cytofix/Cytoperm Fixation/Permeabilization Kit (BD Biosciences). For p-STAT3 staining, cells were preactivated by anti-CD3/CD28 (1 *μ*g/mL) for 24 h, fixed with 2% paraformaldehyde for 10 min at 37°C, and permeabilized by adding 100% pre-cold methanol for 30 min on ice. The resulting cells were stained with anti-human CD4 antibody, anti-p-STAT3 antibody (BD Biosciences), or isotype control (BD Biosciences).

### 2.5. ELISA

Concentrations of the indicated cytokines in serum and synovial fluid specimens were measured by various ELISA sets from R&D Systems and eBiosciences, according to the manufacturer's protocols, respectively.

### 2.6. Immunoblot Analysis

Cell lysates preparations were fractionated by SDS-PAGE, transferred to nitrocellulose membranes, and analyzed by immunoblot using specific antibodies to phospho-STAT3 (Cell Signaling Technology). For immunoblot normalization, the same membrane was stripped and reprobed with specific antibodies against STAT3 (Cell Signaling Technology).

### 2.7. RNA Isolation and Real-Time PCR

Total RNA was isolated from cell pellets using RNeasy Mini Kit (Qiagen) and first strand cDNA was synthesized using Sensiscript RT Kit (Qiagen) according to the manufacturer's instructions. mRNA expression of* RORC* and* GAPDH* was determined by real-time PCR using SYBR Green master mix (Applied Biosystems).* RORC* forward primer sequence was 5′-ACCGATGTGGACTTCGTTTTG-3′ and reverse primer sequence was 5′-CGGTGTGCTGCGGAAACT-3′.* GAPDH* forward primer sequence was 5′-GTGAAGGTCGGAGTCAACG-3′ and reverse primer sequence was 5′-TGAGGTCAATGAAGGGGTC-3′. Thermocycler conditions are comprised of initial holding at 50°C for 2 min and subsequently at 95°C for 10 min, which was followed by a two-step PCR program that consists of 95°C for 15 s and of 60°C for 60 s for 40 cycles. Data were collected and analyzed on an ABI Prism 7500 sequence detection system (Applied Biosystems). The* GAPDH* gene was used as an endogenous control for normalization. All values were expressed as fold increase or decrease relative to the expression of* GAPDH*. The expression level of* RORC* in a given sample was represented as the 2^−ΔCt^, where ΔCt = [Ct (*RORC*)] – [Ct (*GAPDH*)].

### 2.8. Statistics

A Student's* t*-test was used to analyze the differences between the groups. One-way ANOVA was initially performed to determine whether an overall statistically significant change existed before using the two-tailed paired or unpaired Student's* t*-test. Results are presented as the mean ± SEM. *P* < 0.05 was considered as statistically significant.

## 3. Results

### 3.1. Cytokines Involved in Promoting Th17 Cell Differentiation in Synovial Fluid from RA Patients

**Figure 1:**
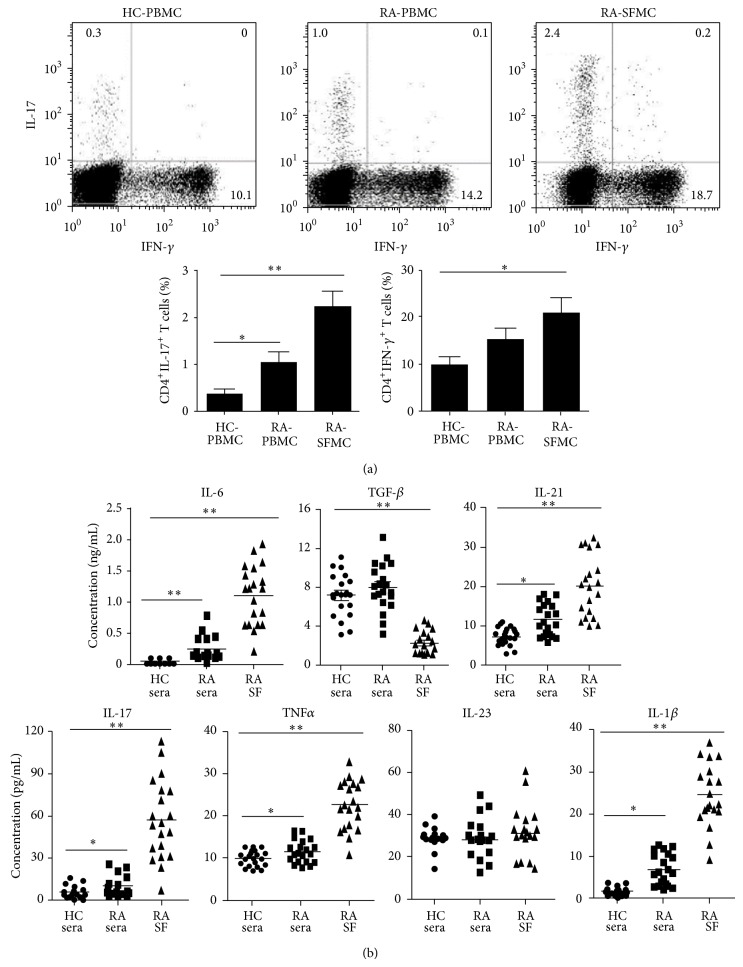
Increased percentage of Th17 and Th1 cells in RA synovial fluid. (a) Percentage of Th1 and Th17 cells was analyzed in CD4 subset from PBMC and SFMC of RA patients (RA-PBMC, RA-SFMC) or PBMC of healthy individuals (HC-PBMC) by intracellular staining of IFN-*γ* and IL-17, respectively, using flow cytometry. Data are representative of 20 samples (up row). Bars show the mean ± SEM (down row). (b) Cytokine levels were measured by ELISA in synovial fluid (SF) and serum samples from 20 RA patients. A panel of 20 serum specimens from healthy individuals was included as controls (HC). Lines represent mean concentration values. In all cases, asterisks indicate statistically significant differences between the groups (^*^ *P* < 0.05, ^**^ *P* < 0.01).

We first measured the number of circulating Th17 cells in the peripheral blood and synovial fluid from RA patients by flow cytometry. As depicted in Figure 1(a), the expression of IL-17 and IFN*γ* was markedly increased in SFMC (2.23 ± 0.33 and 19.76 ± 2.07, resp.) derived from RA patients by intracellular staining compared to that of PBMC (1.04 ± 0.24 and 15.41 ± 2.36, resp.) or control PBMC (0.37 ± 0.10 and 10.91 ± 3.31, resp.). Therefore, the increased frequencies of circulating Th17 and Th1 cells could contribute to inflammation in RA patients.

**Figure 2:**
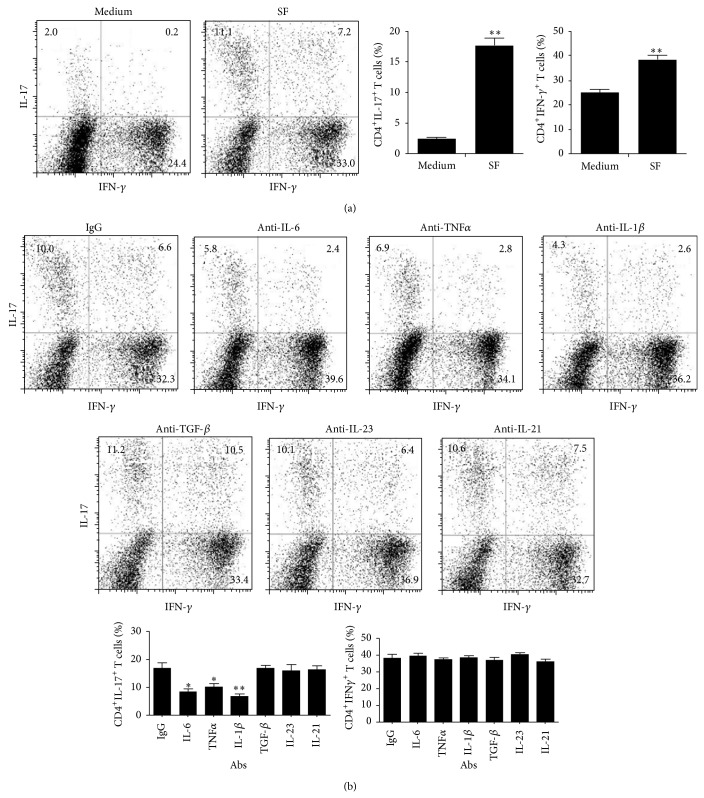
Enhanced Th17 cell differentiation by IL-6, IL-1*β*, and TNF*α* in synovial fluid. (a) PBMC preparations from healthy individuals were stimulated with anti-CD3/CD28 antibodies in the presence of the RA SF at dilution of 1 : 8 and intracellular staining of IFN-*γ* and IL-17 was detected using flow cytometry. Data are representative of at least five separated experiments (left panel) and bars show the mean ± SEM (right panel). ^**^ *P* < 0.01, versus medium control. (b) PBMC preparations from healthy individuals were stimulated with anti-CD3/CD28 antibodies for 96 h, in the presence of RA SF pretreated with indicated antibodies. Percentage of Th1 and Th17 cells was analyzed. Data are representative of at least five separated experiments (upper and middle panels) and bars show the mean ± SEM (lower panel). ^*^ *P* < 0.05 and ^**^ *P* < 0.01 versus IgG control.

As cytokines could promote Th17 cell differentiation, so next we measured a panel of cytokines known to influence Th17 cell differentiation. Blood and synovial fluid specimens were obtained from a group of patients with RA and analyzed for cytokine profile using serum samples from age-matched healthy individuals as controls. With the exception of TGF*β* and IL-23, the levels of the indicated proinflammatory cytokines were all markedly elevated in synovial fluid and sera of RA patients compared to those of healthy individuals (Figure 1(b)). Synovial fluid derived from RA patients showed a significant effect in promoting differentiation of Th17 and Th1 cells in control PBMC preparations (Figure 2(a)). Furthermore, Th17 cell differentiation was blocked by the addition of neutralizing antibodies to TNF*α*, IL-6, and IL-1*β*, respectively, but not antibodies against TGF*β*, IL-21, or IL-23 receptors (Figure 2(b)).

**Figure 3:**
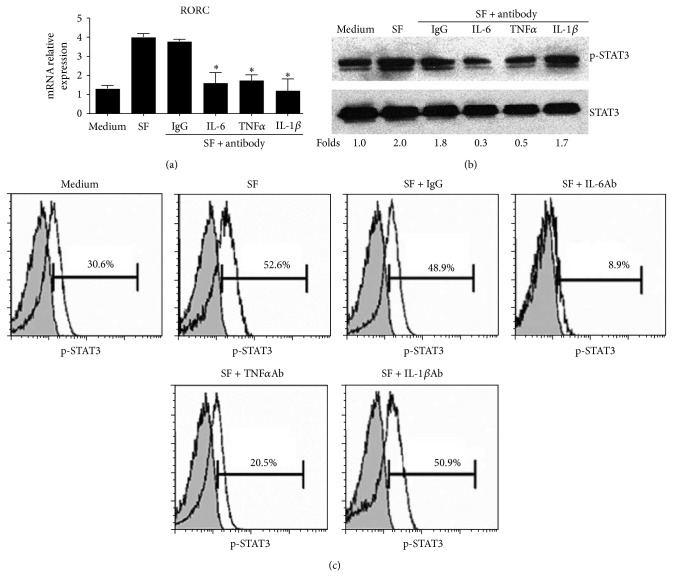
Enhanced Th17 cell differentiation by IL-6, IL-1*β*, and TNF*α* in synovial fluid through the STAT3 and RORC pathway. (a) PBMC preparations from healthy individuals were stimulated with anti-CD3/CD28 antibodies in the presence of the RA SF at dilution of 1 : 8 pretreated with indicated antibodies and mRNA abundance of RORC was determined by real-time PCR after 18 h culture. Bars show the mean ± SEM. ^*^ *P* < 0.05 versus IgG control. (b and c) PBMC preparations from healthy individuals were stimulated with anti-CD3/CD28 antibodies in the presence of RA SF pretreated with indicated antibodies for 24 h. The p-STAT3 and STAT3 levels were detected by western blot (b), or the p-STAT3 expression was analyzed by flow cytometry gated on CD4^+^ T cells (c). Data are representative of at least five separated experiments.

STAT3 phosphorylation and RORC expression levels are critical for Th17 cell differentiation. When the PBMC preparations derived from healthy individuals were treated with RA-SF, the RORC expression was increased and significantly decreased when adding the antibodies to IL-6, IL-1*β*, and TNF*α*, respectively (Figure 3(a)). The STAT3 phosphorylation levels of control PBMC treated with RA-SF were significantly increased compared with medium group, which was blocked by the addition of neutralizing antibodies to TNF*α* and IL-6, respectively, by western blot (Figure 3(b)). Similar result was also observed by flow cytometry when gated on CD4^+^ T cells (Figure 3(c)). Taken together, cytokines of TNF*α*, IL-6, and IL-1*β* in RA-SF played a critical role in promoting Th17 cell differentiation in rheumatoid synovial joints through the STAT3 and/or RORC pathway.

### 3.2. Th17 Cell Responses Are Suppressed by Anti-TNF*α* via Inhibition of Monocytes Derived IL-6 and IL-1*β* Production

**Figure 4:**
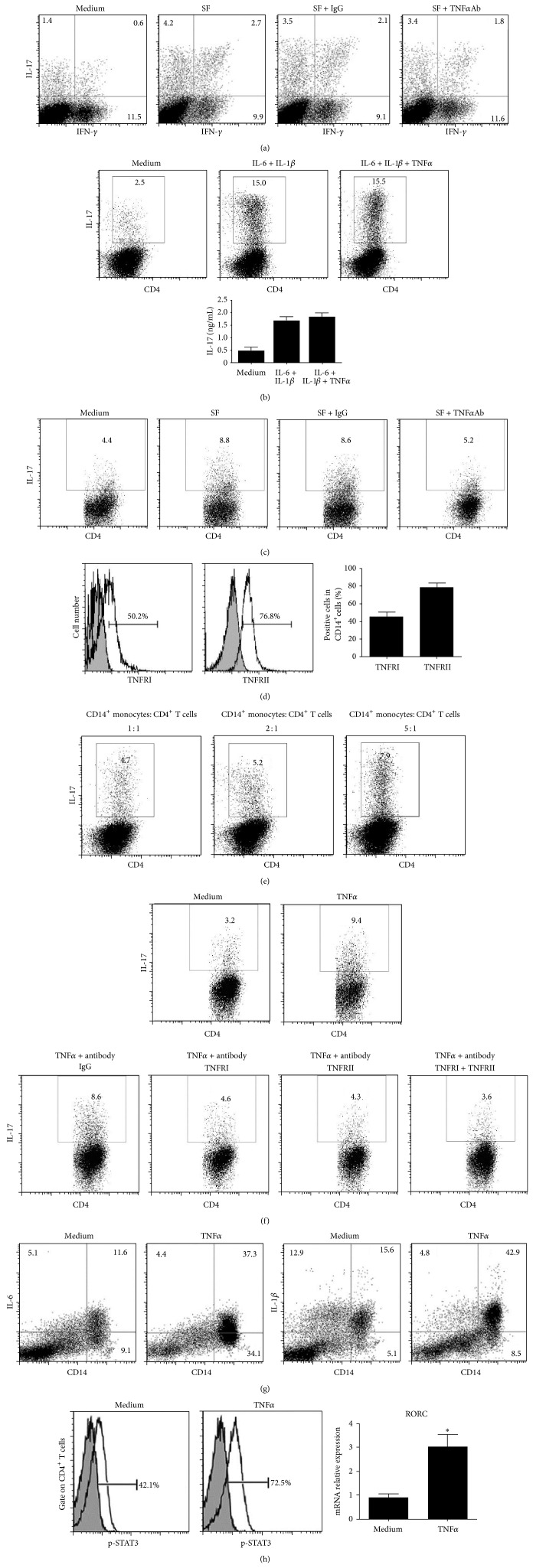
Role of TNF*α* in the Th17 cell differentiation through IL-6 and IL-1*β* in CD14^+^ monocytes. (a) CD4^+^ T cells purified from PBMC of healthy individuals were cultured with RA SF (dilution 1 : 8) in the presence or absence of the indicated antibodies in presence of anti-CD3 and anti-CD28 for 5 days. Percentage of Th1 and Th17 cells was analyzed in CD4 subset by flow cytometry. (b) FACS-sorted CD4^+^CD45RA^+^CD25^−^ naïve T cells from healthy individuals were stimulated with the indicated conditions for 5 days and intracellular staining of IL-17 was analyzed by flow cytometry (up row) and the supernatant was collected for IL-17 detection by ELISA (down row). (c) CD14^+^ monocytes purified from PBMC of healthy individuals were cultured overnight with RA SF in the presence or absence of the indicated antibodies. The resulting cells were cocultured with CD4^+^ T cells derived from the same donor in presence of anti-CD3 and anti-CD28 for 5 days. Percentage of Th17 cells was analyzed in CD4 subset by flow cytometry. (d) TNFRI and TNFRII expression levels were detected by flow cytometry gated on the CD14^+^ monocytes from the healthy individuals. Bars show the mean and SEM. (e) Purified CD4^+^ T cells derived from healthy individuals were cocultured with CD14^+^ monocytes with different ratios in the presence of TNF*α* (50 ng/mL) for 5 days and percentage of Th17 cells was analyzed in CD4 subset by flow cytometry. (f) Purified CD4^+^ T cells derived from healthy individuals were cocultured with CD14^+^ monocytes in the presence of TNF*α* and the indicated antibodies for 5 days. Percentage of Th17 cells was analyzed in CD4 subset by flow cytometry. (g) Purified CD4^+^ T cells and CD14^+^ monocytes from healthy individual were cocultured with TNF*α* or not. After being cultured for 5 days, percentage of IL-6 or IL-1*β* producing cells was analyzed in CD14^+^ cells by flow cytometry. (h) In the same experiment setting as in (g), STAT3 phosphorylation in the CD4^+^ T cell population was analyzed by flow cytometry (left panel) after 24 h culture and mRNA abundance of RORC was determined by real-time PCR (right panel) after 18 h culture. Bars show the mean ± SEM. ^*^ *P* < 0.05 versus medium control.

Interestingly, treatment of RA-SF with TNF*α* antibody had no effect on differentiation of purified CD4^+^ T cells to Th17 cells (Figure 4(a)). Also, TNF*α* had no significantly effects on the differentiation of Th17 cells from the CD4^+^CD45RA^+^CD25^−^ naive T cells (Figure 4(b)). These data suggested that TNF*α* could not promote Th17 cell differentiation directly. We thus examined the possibility that the pro-Th17 effect of TNF*α* was mediated through monocytes. As shown in Figure 4(c), coculture of purified CD4^+^ T cells with CD14^+^ monocytes treated with RA-SF led to increased Th17 cell differentiation, which was blocked by TNF*α* antagonism. So we further explored how the monocytes were regulated by the action of TNF*α* signalling. Data show that monocytes express TNF*α* receptor I and receptor II (Figure 4(d)), and coculture of purified CD4^+^ T cells with increased ratio of CD14^+^ monocytes in the presence of TNF*α* led to increase of Th17 cell differentiation (Figure 4(e)), which was blocked by TNF*α* receptor antagonism (Figure 4(f)). Consistently, TNF*α* induced increased production of IL-6 and IL-1*β* in CD14^+^ monocytes (Figure 4(g)) and, more importantly, increased STAT3 phosphorylation and RORC expression in T cells (Figure 4(h)), which correlated with Th17 cell differentiation. These results indicate that proinflammatory cytokine TNF*α* can promote Th17 cell differentiation in active RA patients via enhancement of monocytes derived IL-6 and IL-1*β* production, and the signalling pathway is probably through TNFRI and TNFRII which are both expressed on the surface of monocytes.

### 3.3. Percentage of Th17 Cells Decreased in RA Patients after Infliximab Treatment

**Figure 5:**
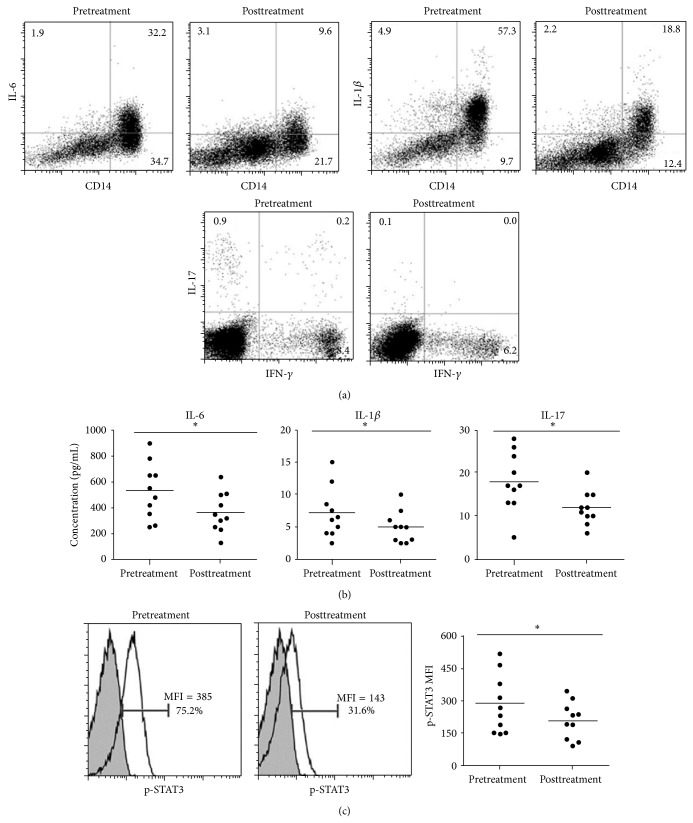
Decreased Th17 cell differentiation after anti-TNF*α* treatment in RA patients. A group of RA patients (*n* = 10) were treated with infliximab. Blood specimens were obtained at baseline or 22 weeks after treatment. (a) The percentage of Th1 and Th17 cells in CD4^+^ T cells and that of IL-6 and IL-1*β* producing cells in CD14^+^ cells were determined by flow cytometry. (b) The concentration of IL-6, IL-1*β*, and IL-17 in sera from the pretreatment or posttreatment of RA patients. (c) The phosphorylation of STAT3 in CD4^+^ T cells from the same specimens was analyzed by flow cytometry. The open or shaded areas represent p-STAT3 antibody staining or isotype control, respectively. The shown data are representative of ten paired PBMC specimens (left panel). MFI: mean fluorescent intensity. Line represents mean MFI values (right panel). In all cases, asterisks indicate statistically significant differences between the two groups (^*^ *P* < 0.05).

Furthermore, the observed roles of Th17 cell differentiation were further evaluated in the blood specimens derived from a group of ten RA patients before and after treatment with an approved TNF*α* antagonist infliximab. There was significant reduction in the percentage of CD14^+^ monocytes producing IL-6 or IL-1*β* and CD4^+^ Th17 cells by flow cytometry (Figure 5(a)), which was confirmed by ELISA (Figure 5(b)). Consistently, the STAT3 phosphorylation was decreased after the patients received the infliximab treatment (Figure 5(c)). The data was consistent with the reported paper that anti-TNF*α* therapy in patients with rheumatoid arthritis decreases Th17 cell populations [30].

## 4. Discussion

Our study reveals a novel role of TNF*α* in promoting differentiation of pathogenic Th17 cells in RA synovium. This is supported by the effect of TNF*α* on Th17 cell differentiation in vitro and by the blocking effect of TNF*α* antibody in experiments involving RA synovial fluid. These findings were further confirmed in RA patients treated with a TNF*α* antagonist as evidenced by markedly decreased IL-17 production and Th17 cell differentiation. Importantly, the observed effect of TNF*α* on Th17 cell differentiation is not directed towards Th17 cells but is mediated through increased phosphorylation of STAT3 in T cells by promoting IL-6 production in monocytes/macrophages. TNF*α*-treated monocytes and not control monocytes are shown to effectively promote Th17 cell differentiation. It should be mentioned that IL-6 may not be the only cytokine involved in the observed effect of TNF*α* in Th17 cell differentiation as it also induces the production of other proinflammatory cytokines, such as IL-1*β* known to promote Th17 cell differentiation. Activation of monocytes from RA patients treated with infliximab was diminished compared to those before treatment, which may reflect suppression of monocytes by anti-TNF*α* leading to a reduction in IL-6 and IL-1*β* and suppression of Th17 cells. In addition, fresh T cells from RA patients expressed high levels of the phospho-STAT3, whereas fresh T cells from patients being treated with infliximab expressed low levels of phospho-STAT3. Correlation of TNF*α* antagonism with reduced Th17 cell responses was also reported in other human autoimmune conditions [31, 32].

Th17 cells constitute a third subset of effecter helper T cells and the effector functions of Th17 cells are distinct from those of Th1 and Th2 cells. Th17 cells and IL-17 play a critical role in the pathogenic mechanisms in an animal model of CIA. Previous studies have analyzed Th17 cells in RA patients receiving anti-TNF therapy (infliximab or adalimumab) with contradictory results and these could be attributed to differences in baseline characteristics of patients and the different responses to the anti-TNF*α* therapy [33–35]. Our data show a reduction in the Th17 subset after infliximab administration. This decrease could be explained by the inactivation of the monocytes after the blockade of TNF*α*, thus producing less IL-6 and IL-1*β* to differentiate into Th17 cells. Other possible explanations for the decrease in the Th17 subpopulation could be an anti-TNF-mediated induction or recovery of cell populations capable of regulating this pathogenic response. It has been reported that CD4^+^ T cells exposed to infliximab could convert into regulatory T cells [36] and they have been reported to suppress Th17 cells in an inflammatory setting [37]. Expansion of Treg cells could be at the expense of Th1 or Th17 cells, when differentiating from naïve CD4^+^ T cells. There are some controversies over the role of IL-6 in the in vitro differentiation of Th17 cells. McGovern et al. report that Th17 responses can be suppressed by Treg cells from RA patients who respond to treatment with adalimumab, via the control of monocyte-derived IL-6 production [30]. But Evans et al. found that IL-6 is independent on Th17 cell differentiation when the monocytes were stimulated with LPS in vitro system [22]. The different role of IL-6 in Th17 cell differentiation may be influenced by the different culture system in vitro, such as stimulated monocytes with LPS or cultured with Treg cells or not. So in addition to cytokine signals, cell-derived signals like costimulation or adhesion may be required for the generation or expansion of Th17 cells.

The study reveals a novel mechanism of action for anti-TNF*α* treatment in RA. It is conceivable that TNF*α* antagonism offers significant advantage in the treatment of RA. Results obtained from recent clinical trials involving anti-TNF*α* agents are consistent with this notion [38]. In addition, the data collectively support the role of IL-6 and IL-1*β* in RA as downstream mediators of TNF*α* for the induction of Th17 cell differentiation and are in an agreement with the results of reported clinical studies involving direct targeting IL-6 receptor or IL-1*β* in RA patients [39–42].  Alzabin et al. reported that an incomplete response of inflammation arthritis to anti-TNF*α* is associated with Th17 pathway [35]. Thus, blocking Th17 cell differentiation may be a very important treatment strategy in RA patients especially those who cannot response to the anti-TNF*α*. Taken together, the study provides new evidence supporting the critical roles of TNF*α* in the pathogenic Th17 cells in RA and perhaps in other autoimmune pathologies where TNF*α* is the key proinflammatory mediator.

## Abbreviations

SF: Synovial fluid
SFMC: Synovial fluid mononuclear cells.
